# Two multi-fragment recombination events resulted in the β-lactam-resistant serotype 11A-ST6521 related to Spain^9V^-ST156 pneumococcal clone spreading in south-western Europe, 2008 to 2016

**DOI:** 10.2807/1560-7917.ES.2020.25.16.1900457

**Published:** 2020-04-23

**Authors:** Aida González-Díaz, Miguel P Machado, Jordi Càmara, José Yuste, Emmanuelle Varon, Miriam Domenech, María Del Grosso, José María Marimón, Emilia Cercenado, Nieves Larrosa, María Dolores Quesada, Dionisia Fontanals, Assiya El-Mniai, Meritxell Cubero, João A Carriço, Sara Martí, Mario Ramirez, Carmen Ardanuy

**Affiliations:** 1 Microbiology Department, Hospital Universitari Bellvitge, IDIBELL-UB, L’Hospitalet de LLobregat, Spain; 2 Research Network for Respiratory Diseases (CIBERES), ISCIII, Madrid, Spain; 3 Institute of Microbiology, Institute of Molecular Medicine, Faculty of Medicine, University of Lisbon, Lisbon, Portugal; 4 Pneumococcal Reference Laboratory, Centro Nacional de Referencia, ISCIII, Madrid, Spain; 5 National Reference Centre for Pneumococci, Centre Hospitalier Intercommunal de Créteil, Créteil, France; 6 Infection Diseases Department, Istituto Superiore di Sanità, Rome, Italy; 7 Biodonostia, Infectious Diseases Area, Respiratory Infection and Antimicrobial Resistance Group, Osakidetza Basque Health Service, Donostialdea Integrated Health Organisation, Microbiology Department, San Sebastian, Spain; 8 Clinical Microbiology and Infectious Disease Department, Hospital General Universitario Gregorio Marañón, Madrid, Spain; 9 Microbiology Department, Hospital Universitari Vall d’Hebron, UAB, Barcelona, Spain; 10 Microbiology Department, Clinical Laboratory North Metropolitan Area, Hospital Universitari Germans Trias i Pujol, UAB, Badalona, Spain; 11 Microbiology Department, Hospital Universitari Parc Taulí, Sabadell, Spain; 12 Department of Pathology and Experimental Therapeutics, School of Medicine, University of Barcelona, Barcelona, Spain

**Keywords:** *Streptococcus pneumoniae*, Multi-fragment recombination, CC156, vaccine escape

## Abstract

**Background:**

The successful pneumococcal clone Spain^9V^-ST156 (PMEN3) is usually associated with vaccine serotypes 9V and 14.

**Aim:**

Our objective was to analyse the increase of a serotype 11A variant of PMEN3 as cause of invasive pneumococcal disease (IPD) in Spain and its spread in south-western Europe.

**Methods:**

We conducted a prospective multicentre study of adult IPD in Spain (2008–16). Furthermore, a subset of 61 penicillin-resistant serotype 11A isolates from France, Italy, Portugal and Spain were subjected to whole genome sequencing (WGS) and compared with 238 genomes from the European Nucleotide Archive (ENA).

**Results:**

Although the incidence of serotype 11A in IPD was stable, a clonal shift was detected from CC62 (penicillin-susceptible) to CC156 (penicillin-resistant). By WGS, three major 11A-CC156 lineages were identified, linked to ST156 (n = 5 isolates; France, Italy and Portugal), ST166 (n = 4 isolates; France and Portugal) and ST838/6521 (n = 52 isolates; France, Portugal and Spain). Acquisition of the 11A capsule allowed to escape vaccine effect. AP200 (11A-ST62) was the donor for ST156 and ST838/6521 but not for ST166. In-depth analysis of ST838/6521 lineage showed two multi-fragment recombination events including four and seven fragments from an 11A-ST62 and an NT-ST344 representative, respectively.

**Conclusion:**

The increase in penicillin-resistant serotype 11A IPD in Spain was linked to the spread of a vaccine escape PMEN3 recombinant clone. Several recombination events were observed in PMEN3 acquiring an 11A capsule. The most successful 11A-PMEN3 lineage spreading in south-western Europe appeared after two multi-fragment recombination events with representatives of two major pneumococcal clones (11A-ST62 and NT-ST344).

## Introduction

*Streptococcus pneumoniae* is an important human pathogen that usually colonises the upper respiratory tract. Pneumococci can invade sterile sites causing different invasive pneumococcal diseases (IPD) such as bacteraemic pneumonia, meningitis or primary bacteraemia. Furthermore, pneumococci cause other non-invasive diseases such as acute otitis media in children or acute exacerbations of COPD in adults [1,2]. The polysaccharide capsule, which presents one of more than 97 serotypes, is the main virulence factor and its diversity has been associated with differences in invasiveness and mortality. Pneumococcal conjugated vaccines (PCV) include the serotypes more commonly found among IPD. In Spain, three conjugate vaccines had been licensed: the first one in 2001, PCV7, targeted serotypes 4, 6B, 9V, 14, 18C, 19F and 23F; the second in 2009, PCV10, added serotypes 1, 5 and 7F and PCV13 in 2010 added serotypes 3, 6A and 19A [3]. The natural ability of *S. pneumoniae* to undergo genetic transformation allows pneumococci to change the capsule (capsular switching) which allows them to escape vaccine pressure. The emergence of new recombinant clones along with serotype replacement has changed the serotype distribution after the introduction of paediatric vaccination [4,5]. Among them, an increase in serotype 11A, not included in PCV13, has been reported worldwide [1,4,6,7]. Many studies suggest that serotype 11A has a low invasive potential [5] being present mainly in child carriers [8] and patients with chronic obstructive pulmonary disease (COPD) [9]. Serotype 11A has classically been linked to the antibiotic-susceptible lineage CC62 [10], with the exception of some macrolide-resistant isolates with the M phenotype (resistance to 14- and 15-membered ring macrolides) [11]. In 2005, the emergence of a penicillin- and amoxicillin-resistant serotype 11A lineage related to CC156 was identified [10]. In our setting, serotype 11A isolates related to the CC156 clone were the leading cause of β-lactam resistance in the years 2015 and 2016 [12]. Nowadays, in Spain, there are two main serotype 11A sequence types (ST) derived from CC156: ST838, first detected in 2005, which is a single locus variant (SLV) of ST156; and ST6521 which is a double locus variant (DLV) of ST156 emerged in 2009 [10]. These STs are related to clonal complex CC156 also known as the Spain^9V^-ST156 clone (PMEN3). This clone, originally associated with serotype 9V, has been related to capsular switching since the 1990s when the acquisition of the capsule 14 led to a worldwide spread of this serotype. In fact, most of serotype 14 isolates causing IPD in the pre-PCV era were related to the PMEN3 recombinant lineage [13,14]. Besides serotype 14, PMEN3 recombined with other serotypes showing a high capacity to exchange the capsular locus, resulting in the evasion of the vaccine effects [15]. 

In this study, we firstly studied the increase of β-lactam-resistant serotype 11A pneumococci as the cause of adult IPD in the framework of a multicentre study in Spain. Secondly, using whole genome sequencing (WGS), we analysed the spread of the recombinant clone S11A-ST6521 including neighbouring countries in south-western Europe which also detected penicillin-resistant serotype 11A isolates.

## Methods

### Study design and bacterial characterisation

This study was initially conducted in the framework of a laboratory-based multicentre study of adult (≥ 18 years-old) IPD patients, involving six Spanish hospitals. An IPD episode was defined as the isolation of *S. pneumoniae* from a normally sterile body fluid and only one isolate per episode was included [16]. We included IPD episodes caused by serotype 11A from 2008 to 2016.

Serotyping by dot blot assay or Quellung reaction was done at the Spanish Reference Laboratory for Pneumococci (SRLP). The antibiotic susceptibility to seven antimicrobials (amoxicillin, cefotaxime, cotrimoxazole, erythromycin, levofloxacin, penicillin and tetracycline) was tested by microdilution following the European Committee on Antimicrobial Susceptibility Testing (EUCAST) guidelines [17]. Genotyping was performed by pulse field gel electrophoresis (PFGE) and/or multilocus sequence typing (MLST) as described previously [18,19].

### Whole-genome sequencing and assembly

To evaluate the spread of the serotype 11A multidrug-resistant clone, a total of 61 penicillin-resistant (≥ 0.12 mg/L) serotype 11A isolates were selected for WGS. Of these, 21 were collected from the multicentre study and the remaining 40 were obtained from the Reference Laboratory for Pneumococci or Pneumococcal Reference Groups from France (n = 17), Italy (n = 4), Portugal (n = 4) and Spain (n = 16). Metadata including demographic characteristics, WGS-derived data and antibiotic minimum inhibitory concentrations (MIC) are summarised in Supplementary Table S1. A diagram of the study design and a flowchart of the bioinformatic analysis are also summarised in Supplementary Figure S1.

Bacteria were grown overnight in 5% sheep blood agar at 37ᵒC + 5% CO_2_. DNA was extracted using the QIAamp DNA Blood Mini Kit (Qiagen, Hilden, Germany) and quantified using the QuantiFluor dsDNA System (Promega, Wisconsin, United States (US)). Illumina paired-end libraries (2 × 150 bp) were prepared with the Nextera XT kit and sequenced on Illumina MiSeq Platform (Illumina Inc., San Diego, US). Read quality assessment and genome assembly was done using the INNUca v3.2 pipeline (https://github.com/B-UMMI/INNUca) through ummidock/innuca:3.2–01. Firstly, a quality control of the reads was performed using FastQC v0.11.5 (http://www.bioinformatics.babraham.ac.uk/projects/fastqc/) and reads were cleaned and trimmed with Trimmomatic v0.36 [20]. The genome was assembled using SPAdes v3.11.0 [21] and subsequently polished using Pilon v1.18 [22]. The in silico MLST was determined using Seemann’s MLST v2.11 (https://github.com/tseemann/mlst). The final assemblies were annotated by Prokka v.1.12 [23] through ummidock/prokka:1.12 Docker image. Reads were deposited at the European Nucleotide Archive (ENA) with accession numbers summarised in Supplementary Table S1.

### Comparative analysis by whole genome sequencing

#### Molecular antibiotic resistance mechanisms

The in silico analysis of gene mutations involved in antibiotic resistance (*pbp1a, pbp2x, pbp2b, parC, parE, gyrA, folA* and *folP*) was done manually using Geneious R9 (Biomatters, Auckland, New Zealand) and the R6 genome (NC_003098) as reference. The acquired resistance mechanisms were screened using Abricate v0.8.0 (https://github.com/tseemann/abricate) through flowcraft/abricate:0.8.0–3 Docker image for the Comprehensive Antibiotic Resistance Database (CARD) [24] and ResFinder [25] databases.

#### Phylogenetic analysis

To better explore the diversity and relationship with other pneumococci with related serotype and ST to the penicillin-resistant 11A isolates included in this study, 47,529 available *S. pneumoniae* genomes deposited in ENA were downloaded on 29 October 2018 using getSeqENA v1.3 (https://github.com/B-UMMI/getSeqENA) with Aspera Connect v3.7.2.141527 (https://asperasoft.com/software/clients/connect/). In case of large fastq file sizes, they were first downsampled for an estimated depth of coverage of 100 × with the sample_fastq script commit 01ac0db available at https://github.com/jacarrico/sample_fastq and making use of Seqtk v1.2-r94, (https://github.com/lh3/seqtk). The serotype was in silico deduced using Seroba v1.0.1 [26]. Among the 47,529 available genomes, we selected 238 with serotypes 9V or 11A and with one of the following MLST: ST156, ST166, ST838 or ST6521 (Supplementary Table S2). Phylogenetic analysis was performed by constructing an assembly-based core genome-single nucleotide polymorphism (SNP) phylogenetic tree with the default parameters of *Parsnp* from the Harvest suite [27] with the exception of parameter ‘x’ which identifies and removes recent recombination using PhilPack [28]. Isolate ERR2681167 was used as reference. Phylogenetic tree visualisation was done using Microreact [29].

#### Capsular operon analysis

Three contiguous regions were analysed: region 1 (*pbp2x* to *dexB),* region 2 (capsular locus) and region 3 (*aliA* to *pbp1a*). Because the capsular operon is flanked by transposases, which are difficult to assemble using the small reads generated by Illumina methodology, these regions were located in different contigs, so it was not possible to analyse the whole region from *pbp2x* to *pbp1a*. A BLASTn search on the National Center for Biotechnology (NCBI) website (https://blast.ncbi.nlm.nih.gov/Blast.cgi) was performed to determine which deposited fully closed genome presented the highest identity with the isolates included in the study. In addition, region 2 was compared with reference capsular operons (1813/39–11A (CR931653) and 980/60–9V (CR931648)) previously described [30].

#### Identification of recombination regions

To determine the genomic differences between ST838 and ST6521 isolates, a maximum-likelihood phylogenetic tree was constructed with the 68 isolates belonging to these two ST, 52 from this study and 16 from ENA, using RaxML [31]. The 9V-ST838 (ERR2303060) draft assembled genome was concatenated by mapping the contigs against 4041STDY6836167 (NZ_LS483448), which is a closed serotype 9V, ST156 genome using Mauve [32]. The 9V-ST838 concatenated genome was used as a reference and the 68 reads belonging to lineage 3 isolates were mapped using Snippy 3.1 (https://github.com/tseemann/snippy). An assembly-free core-SNP alignment was done with Snippy’s core module (snippy-core). The alignment was used to identify the recombinant regions through the evaluation of the density of base substitution using Gubbins v2.3.4 [33] and the results were visualised in Phandango [34]. The allelic variation of fragment exchange in recombination events were manually examined with Geneious R9 and the highest identity with *S. pneumoniae* genomes available on NCBI was explored using BLASTn search (https://blast.ncbi.nlm.nih.gov/Blast.cgi).

#### Core and accessory genome 

The core genes, which are shared by all isolates, and accessory genes of the 68 isolates belonging to ST838 and ST6521 were analysed using Roary v3.12.0 [35], with a minimum percentage identity of 80% for BLASTp. Identification of accessory genes segregating each ST was done by Scoary v1.16.16 [36].

### Ethical statement

This project has been approved by the Clinical Research Ethics Committee of the Hospital de Bellvitge (PR153/18). Written informed consent was not required as this was an observational study with isolates obtained as part of the normal microbiological routine. Patient confidentiality was always protected; all data were anonymised and protected according to national normative.

## Results

### A clonal shift has occurred among serotype 11A isolates causing adult invasive pneumococcal disease in Spain

**Figure 1:**
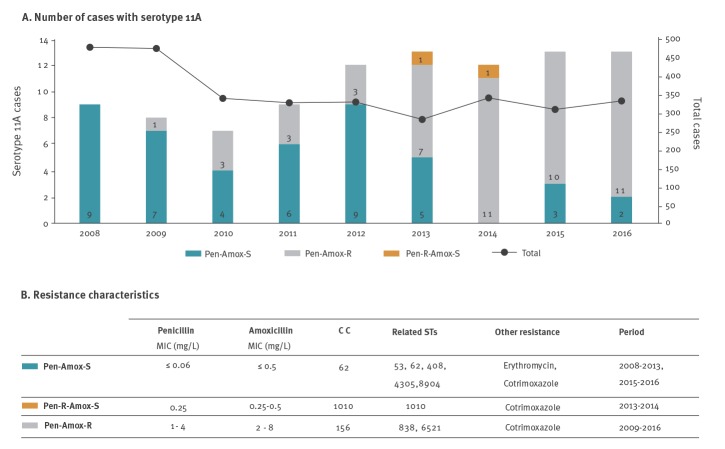
Total and pneumococcal serotype 11A cases, associated clonal complex and resistance, south-western Europe, 2008-2016 (n = 96)

Among 3,200 adult IPD episodes detected during the study period, 96 were caused by serotype 11A isolates. We observed a non-significant increase in the incidence of serotype 11A IPD from 0.24 episodes per 100,000 persons per year in 2008 to 0.36 episodes per 100,000 persons per year in 2016 (p = 0.22; incidence risk ratio = 0.66; 95% confidence interval (CI): 0.37–1.20). However, a clonal replacement linked to an increase in penicillin-resistant isolates was observed. While in 2008, no penicillin-resistant serotype 11A isolates were detected, 11 of 13 IPD episodes due to serotype 11A were caused by penicillin-resistant isolates in 2016 (Figure 1). The PFGE/MLST analysis revealed two major clonal complexes: the penicillin-susceptible CC62 and the penicillin-resistant CC156. Isolates of CC62 were mostly antibiotic-susceptible (including to penicillin) except for several cotrimoxazole- (26 of 44; 59.1%) or erythromycin-resistant (13 of 44; 29.5%) isolates. Macrolide resistance was due to the M phenotype, as previously described among CC62 isolates in Spain [11]. On the other hand, isolates of CC156 showed a common resistance pattern, being penicillin-resistant (MIC range: 1–4 mg/L) and cotrimoxazole-resistant (MIC range: 1/19 – >2/38 mg/L). Moreover, CC156 isolates were mostly amoxicillin-resistant with a MIC range from 2 to 8 mg/L (Figure 1).

Amox: amoxicillin; CC: clonal complex; IPD: invasive pneumococcal disease; MIC: minimum inhibitory concentration; Pen: penicillin; R: resistance; S: susceptible; ST: sequence type.

The recombinant clone ST6521-serotype 11A is spreading in Europe.

#### Whole genome sequencing of penicillin-resistant serotype 11A from south-western Europe

For an in-depth genomic analysis of penicillin-resistant serotype 11A isolates related to CC156, a collection of 61 penicillin-resistant serotype 11A isolates isolated from patients with IPD in France, Italy, Portugal and Spain were subjected to WGS (Supplementary Figure S1). These isolates were recovered from 25 regions of the four European countries: France (n = 9), Italy (n = 2), Portugal (n = 3), and Spain (n = 11) (Supplementary Figure S2).

After in silico MLST analysis, six different ST were found: ST156, ST166 (SLV of 156), ST838 (SLV of ST156), ST6521 (SLV of ST838 and DLV of ST156), a new SLV of ST6521 (ST14589) and a new DLV of ST6521 (ST14590). Supplementary Figure S3 shows the phenotypic (MIC) and genotypic (resistome) profiles of the 11A-CC156 isolates and their association with the ST. The allelic profiles of their penicillin-binding proteins (PBP) are described in Supplementary Table S3, a name was assigned to each allele based on the previous definition by the US Centers for Disease Control and Prevention (CDC) [37,38]. The differences between ST in resistance to β-lactams were linked to changes in PBP. A common PBP1A allele (allele 15) identical to that of PMEN1 (American Type Culture Collection 700669) was shared by all but three isolates (the latter presented allele 7 and a new allele (NEW1) close to allele 96 (96% identity)). We found the changes at the STMK373 motif (T371A allele 15 and T371S in allele NEW1) that had been previously described [15]. All but two isolates presenting allele 15, presented PBP2X allele 18 and a new allele (NEW1) related to allele 36 (98.9% identity). Both alleles differed in only three amino acids and have the 337S**A**MK substitution. Two distinct PBP2B alleles were found segregating lineages ST156 and ST166 from ST838 and ST6521. All isolates presented the 443SSN**A** change in PBP2B. Moreover, isolates with allele 76 had an additional 10 changes between residues 590 and 641 related to increased amoxicillin MIC [15].

Macrolide-resistance was associated with the presence of integrative conjugative elements. All four ST166 isolates carried the Tn*6002* transposon (AY898750) described previously in *S. pneumoniae*. This is a Tn*916*-like structure containing the *tet*(M) gene and the macrolide-aminoglycoside-streptothricin (MAS) element *erm*(B), conferring resistance to tetracycline and to erythromycin and clindamycin, macrolide-lincosamide-streptogramin b (MLS_B_) phenotype, respectively. One 11A-ST6521 isolate carried a mobile element, the *mef*(E) gene in the macrolide efflux genetic assembly (MEGA) element. All isolates were resistant to cotrimoxazole, a characteristic of the Spain^9V^-ST156 (PMEN3) clone. All but one had the I100L amino acid substitution in the dihydrofolate reductase known to confer resistance to trimethoprim. In addition, all isolates had a single insertion (S_62_S**S**Y) or double insertion (S_62_S**YS**Y; in ST166 isolates) in the dihydropteroate synthase, known to confer resistance to sulfamethoxazole.

#### Different capsular switching events identified among the major serotype 11A lineages

**Figure 2:**
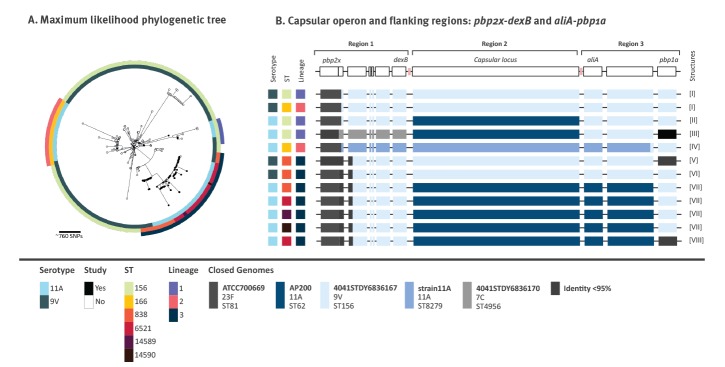
Capsular switching and recombination breakpoints, pneumococcal isolates, south-western Europe

BLAST: basic local alignment search tool; ENA: European Nucleotide Archive; MLST: multilocus sequence typing; NCBI: National Center for Biotechnology Information; SNP: single nucleotide polymorphism; ST: sequence type.

Of the 238 selected genomes from ENA, 198 were serotype 9V (n = 183 ST156, n = 2 ST166 and n = 13 ST838) and the remaining 40 were serotype 11A (n = 5 ST156, n = 32 ST166 and n = 3 ST6521). A phylogenetic tree was constructed using these 238 genomes and the 61 genomes of the present study (Figure 2A, Supplementary Table S2). Three major lineages, including both 9V and 11A isolates, were defined including one or two closely related ST: lineage 1 (ST156), lineage 2 (ST166) and lineage 3 (ST838 and ST6521). To identify putative recombination events in the capsular locus we used all isolates, except in lineage 1, which accounted for the highest number of genomes (n = 188). In order to simplify the analysis of the capsular switching event in this lineage we selected all five 11A-ST156 isolates from this study and the four phylogenetically closest 9V-ST156 isolates (Supplementary Table S2).

Panel A: The maximum likelihood phylogenetic tree includes the 61 sequenced strains and 238 strains of serotypes 9V and 11A for which genomes were available in ENA and which belonged to ST related to CC156 (ST156, ST166, ST838 or ST6521). The colour of each circle represents different characteristics of the strains. Outer circle: lineages; middle circle: ST; inner circle: serotypes.

Panel B: The analysis of the capsular operon was done with 61 genomes from this study and a selection of 59 from the 238 ENA genomes. The different structures are numbered I to VIII. Colours are related to the closest fully closed genome on NCBI (indicated in the legend with serotype and MLST). Black regions: areas with identity < 95% with NCBI fully closed genomes after BLAST search; pink squares: non-analysed flanking transposases.

The analysis of the capsular locus and the flanking regions (region 1: *pbp2x*-*dexB*; region 2: capsular locus; region 3: *aliA*-*pbp1a*) in all lineages revealed eight different structures (numbered I to VIII; Figure 2B). Structure definition was based on per cent identity with complete genomes available in NCBI, where sequence stretches with identity lower than 95% were coloured in black. Among serotype 9V, three different structures were found (I, V and VI), the main differences among them were the region downstream of *pbp2x* in structures V and VI, and *pbp1a* in structure V. On the other hand, five different structures (II, III, IV, VII and VIII) were found among serotype 11A isolates. Two of them (II and III) were detected in lineage 1. Structure III presented different *pbp2x* and *pbp1a* genes and a different sequence in region 1, with high identity with the 4041STDY6836167 genome (NZ_LS483448), a serotype 7C-ST4956 isolate. All lineage 2 isolates had structure IV, which was closely related to that of an 11A isolate named strain11A (NZ_CP018838). However, the capsular locus (region 2) of structure IV had only 92% identity with the 11A capsule locus of the reference isolate 1813/39–11A [30]. Structure IV showed high identity (> 99%) with isolate PMP1342 (MF140334.1), a genetic variant of the 11A locus, with the exception of the *wzg* gene (identical to that of the 980/60–9V reference isolate). Finally, all but one of the lineage 3 isolates shared structure VII. In this structure the DNA fragment integrated from AP200 (11A-ST62) included the capsular operon (region 2) and a portion of the region 3 but not the *pbp1A*, which was similar to that of 4041STDY6836167, the 9V-ST156 isolate (Figure 2B). Structure VIII was found in a single isolate and differed from structure VII in the *pbp1a* gene. There were amino acid changes in some genes of the capsular locus in all structures which are summarised in Supplementary Table S4.

#### Several results of recombination events are present in the successful 11A-ST6521 lineage

**Figure 3:**
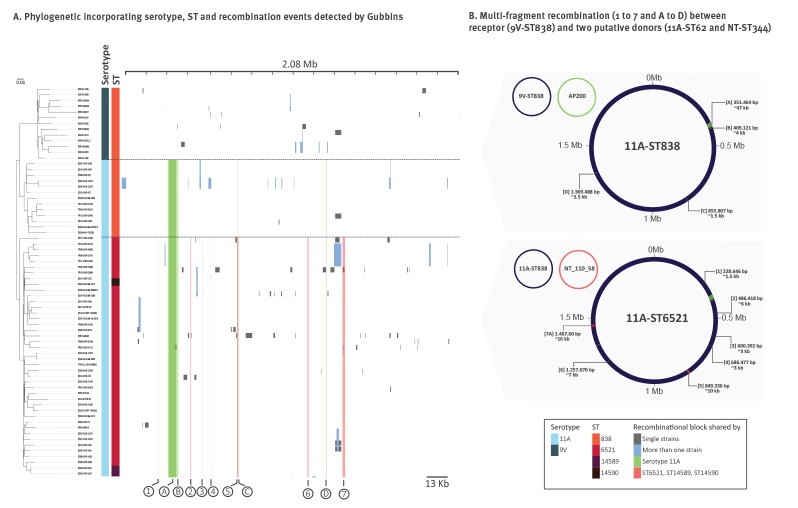
Analysis of recombination in lineage 3 (serotypes 9V and 11A), pneumococcal isolates, south-western Europe (n = 68)

A scan for possible recombination events across the entire genome and identification of the possible donors was done with the 68 isolates of lineage 3, corresponding to the most successful emerging penicillin-resistant serotype 11A isolates. Besides several possible recombinations identified among single isolates or minor clusters, we found two major recombination events (Figure 3). We considered as the donor the isolate which provided the capsule to the new recombinant isolate and as the recipient the isolate which incorporated the capsule sequence in its genetic background. The first recombination event involved a 9V-ST838 isolate as the recipient and a serotype 11A-ST62 isolate related to AP200 (NC_014494) as the donor. In this event, the capsule which allow to escape the vaccine effect (ca 16 kb, included in region A) and three additional regions (B–D) of the AP200-like isolate were incorporated into the 9V-ST838 genome (represented in green in Figure 3). Region A was a ca 50 kb recombination region which on closer analysis appeared to be discontinuous. This region included four segments of the AP200-like isolate (26 kb, 12 kb, 4.5 kb and 0.5 kb) interspersed with two small segments of the 9V-ST838 isolate (2.5 kb and 4 kb), including the *pbp1a* gene. There was an additional 2 kb segment (between the 12 kb and 4.5 kb segments of the AP200-like isolate) different from both isolates which could be characteristic of the actual donor. Region A could therefore have resulted from a multi-fragment sequence integration. On the other hand, the recombination in the B, C and D regions incorporated fragments of ca 4 kb, 1.5 kb and 1.5 kb, respectively. A second multi-fragment recombination event possibly involved the recombinant 11A-ST838 isolate as recipient and a representative of the worldwide disseminated non-typable ST344 lineage (Figure 3). We identified seven fragments with high similarity to the NT-ST344 isolate NT_110_58 (NZ_CP007593) in the recipient genome (11A-ST838). One of these fragments included the *aroE* gene, explaining the variation in the MLST profile.

ST: sequence type.

Panel A: Green blocks: recombination segments for which putative donor is 11A-ST62 (AP200) and which are shared by strains of ST838, ST6521, ST14589 and ST14590; these blocks are labelled with capital letters (A to D). Red blocks: recombination segments which putative donor is NT-ST344 which are shared by strains ST6521, ST14589 and ST14590; these blocks are labelled with numbers (1 to 7). Grey blocks: recombination segments present in a single strain. Light blue blocks: recombination segments shared by small groups of strains.

Panel B. Dark blue: receptor 9V-ST838; green: putative receptor 11A-ST62; red: putative receptor NT-ST344. Coordinates refer to the ones of the genome of strain 4041STDY6836167. The approximate recombining fragment size is indicated.

#### Allelic variation between ST838 and ST6521 in accessory genome and core genes

In a previous study, we showed different behaviours in evasion of host defenses and in biofilm formation between the two major ST of serotype 11A lineage 3 (11A-ST838 and 11A-ST6521) [10]. In lineage 3, core genes represented around 70% of the total genes detected. Among the 1,707 core genes, the region A multi-fragment recombination introduced 25 allelic variants into the recipient genome, in addition to the serotype 11A specific *cps* locus genes which are not part of the core genome. Furthermore, the second recombination event between 11A-ST838 and NT_110_58 incorporated 37 allelic variants into the seven exchanged fragments differentiating 11A-ST6521 from 11A-ST838. Most of the core genes presented just one allele, while others presented two or more alleles. Most of the allelic differences among core genes did not segregate serotypes, ST or sub-lineages, with the exception of the allelic differences incorporated in the recombination events. The 62 allelic variations potentially introduced by recombination are summarised in Supplementary Table S5. The analysis of the accessory genome performed with Scoary revealed one gene that segregated ST6521 from ST838 (p < 0.01). This gene was a Gcn5-related N-acetyltransferase (spnnt_RS02275) which was truncated in the ST838 isolates following to a single nucleotide deletion.

## Discussion

Current prevention of pneumococcal infections is based on vaccination and vaccines are serotype-dependent, offering protection against a subset of known serotypes. Only 13 serotypes are included in the conjugate vaccine with the highest available valency, PCV13; its widespread use changed the worldwide serotype distribution [1]. During a multicentre study, a clonal replacement was observed among invasive pneumococci of serotype 11A (not included in PCV13), associated with the emergence of β-lactam resistance. This new lineage, related to Spain^9V^-ST156, was penicillin- and amoxicillin-resistant hampering the treatment of severe infections. Besides IPD, this amoxicillin-resistant variant of serotype 11A has in Spain caused acute exacerbations of COPD patients [9] and otitis media in children [39], infections for which amoxicillin usually is the first-choice antibiotic therapy.

We analysed by WGS 61 isolates, from Spain and other south-western European countries where penicillin-resistant 11A isolates have also been detected. Through this bioinformatic analysis, we tried to reconstruct the recent dynamics of the Spain^9V^-ST156 clone in south-western Europe. We showed that the 11A-ST6521 lineage was present in France, Portugal and Spain. Two additional 11A lineages (ST156 and ST166) were identified among French, Italian and Portuguese isolates. However, the β-lactam MIC were lower than those of ST838 and ST6521, in which high amoxicillin MIC are related to amino acid changes in the transpeptidase domain of PBP2B (residues 590–641), as described [15].

Two different recombinant structures were detected in lineage 1 (11A-ST156) for which we could not discard a common origin. Probably, there was a first recombination with an AP200-like isolate, followed by a second recombination that incorporated a fragment between *pbp2x* and *dexB* which presents high identity with a 7C-ST1623 isolate. But this was not unequivocally shown by our analysis. Moreover, a second lineage (11A-ST166) was detected in France and Portugal, as well as in several isolates deposited in the ENA, showing yet different recombination events. This lineage presented a variant of the serotype 11A capsular operon previously identified in Fiji [40]. The presence of a double insertion in the dihydropteroate synthase and the Tn*6002* transposon carrying *tet*(M) and *erm*(B) was a hallmark of all four ST166 isolates suggesting that another horizontal DNA transfer event was pivotal in conferring antimicrobial resistance to lineage 2 (11A-ST166). Although the number of isolates included in the present work was low, the detection of this lineage in France and Portugal and its multidrug resistance deserve further surveillance.

Another recombination event involved lineage 3 (11A-ST838 giving rise to 11A-ST6521). Besides capsule and PBPs, other regions involving single isolates, minor or major clusters, accounted for the genetic diversity of this lineage. The presence of changes in a single isolate could suggest a patho-adaptative process in the course of invasive disease that was not enough to confer the ability to interpatient spread [41]. On the other hand, the presence of the same change in most isolates from the same ST suggests that it may have a beneficial fitness effect on their spread.

The careful analysis of ST838 and ST6521 serotype 11A isolates identified two subsequent multi-fragment recombination events. The first one occurred between an AP200-like isolate [42] and a 9V-ST838. The major consequence of this recombination was the capsular switch that allowed this lineage to escape vaccine-induced immunity. However, in a scenario of only vaccine pressure, a spread of 11A-ST62 could have been hypothesised. Therefore, it seems that the genetic characteristics of the Spain^9V^-ST156 clone such as the β-lactam resistance in an area with high-level antibiotic consumption were determinants for its success. Probably both vaccine and antibiotic pressure could favour this drift. Besides the capsular operon, three additional fragments from the putative AP200-like donor were integrated which may have resulted in still unidentified but important phenotypic changes. The second event occurred with an ST344 isolate, a non-encapsulated worldwide disseminated clone identified in nasopharyngeal colonisation and non-invasive infections, particularly conjunctivitis [43,44]. In this recombination, seven fragments of the ST344 representative were acquired by 11A-ST838. Although we could not exclude the possibility that these multi-fragment acquisitions occurred in different events from other pneumococci, our results strongly suggest that the two multi-fragment recombinations occurred with two isolates related to AP200 and NT_110_58. The ability of PMEN3 to acquire multiple large genomic regions has been described previously, with a recombinant having acquired 5.3% of its genome from a PMEN1 representative [45]. Recently, it has been suggested that large recombination events are favoured in environments allowing stable cell-to-cell contact, such as colonisation or biofilm-associated infections [46]. Serotype 11A is currently a major serotype colonising children [7], and the serotype replacement in colonisation which occurred after PCV introduction could have favoured the widespread emergence of these 11A-PMEN3 variants. The incorporation of further genetic material from an ST344 representative could offer clues for the spread of 11A-ST6521. Among this acquired DNA, there are genes involved in biofilm formation, such as *lytB* [47]. Possibly, this second recombination could be behind the higher capacity to form biofilms and to evade the host immune system of the recombinant 11A-ST6521 lineage which was previously described [10]. These traits can represent a patho-adaptive advantage of colonisation and also of causing biofilm-related diseases such as otitis media or acute exacerbations of COPD [9,10], possibly contributing to the resilience of PMEN3 lineages as a cause of pneumococcal disease in the PCV era.

## Conclusion

We describe a clonal shift among serotype 11A isolates causing IPD in Spain and the spread of a recombinant clone through different European countries. The first event allowed the β-lactam resistant clone to remain as cause of pneumococcal diseases and to escape the current vaccine. The second event gave adaptative advantages to cause disease. Probably both vaccine and antibiotic pressure could favour this drift. Further studies are needed to determine how much this lineage has increased and what impact the multidrug resistance of this vaccine escape recombinant clone will have on the therapy of IPD and other pneumococcal diseases in south-western Europe. Moreover, the ability of the Spain^9V^-ST156 clone to evolve, resulting in new recombinant lineages with new serotypes, implies a need to monitor this invasive clone and its possible drifts in the future.

## Supplementary Data

556000 Supplementary_Tables

## Supplementary Data

1498000 Supplementary_Figures
