# Supplementary material for: Two multi-fragment recombination events resulted in the β-lactam-resistant serotype 11A-ST6521 related to Spain9V-ST156 pneumococcal clone spreading in south-western Europe, 2008 to 2016
Source: Euro Surveill. 2020 Apr 23;25(16):1900457. doi: 10.2807/1560-7917.ES.2020.25.16.1900457 (PMC7189650; doi:10.2807/1560-7917.ES.2020.25.16.1900457)
Supplement: Supplementary_Figures [file 19-00457_ARDANUY_Supplementary_Figures.pdf]

"This supplementary material is hosted by Eurosurveillance as supporting information alongside the article **"Two multi-fragment recombination events resulted in the β-lactam-resistant serotype 11A-ST6521 related to Spain^{9V}-ST156 pneumococcal clone spreading in south-western Europe, 2008 to 2016"** on behalf of the authors who remain responsible for the accuracy and appropriateness of the content. The same standards for ethics, copyright, attributions and permissions as for the article apply. Eurosurveillance is not responsible for the maintenance of any links or email addresses provided therein."

Supplementary Figure S1. Study design and Bioinformatic analysis diagram. Schematic representation of flowchart used in this paper. Genomes from the previous multicentric study, genomes from National Reference centres and genomes downloaded from ENA 29-10-2018 are represented. All figures, supplementary material as well programs and pipelines used in bioinformatic analysis are also specificity in the diagram.

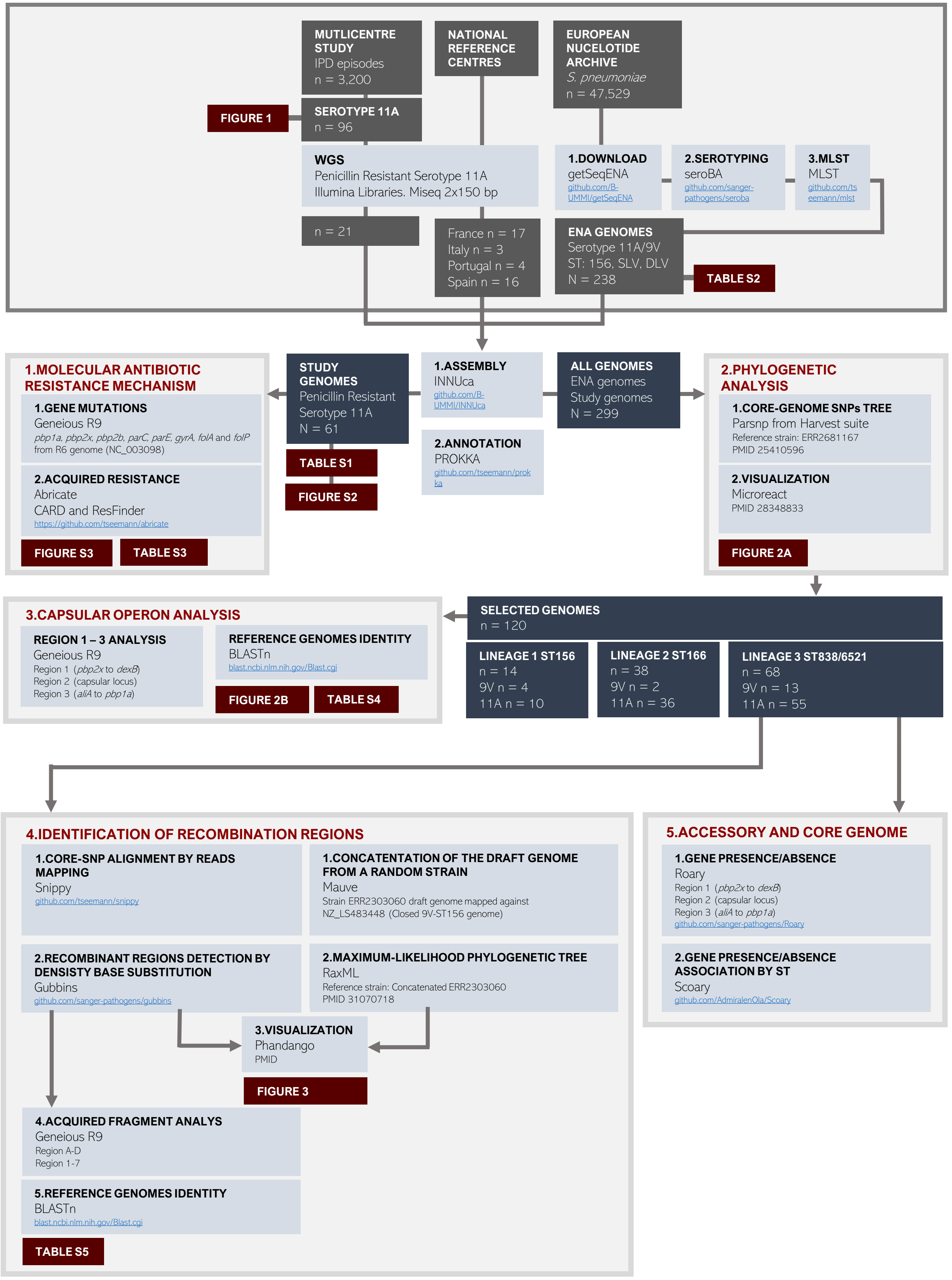
MUTLICENTRE STUDY
IPD episodes
n = 3,200
NATIONAL REFERENCE CENTRES
EUROPEAN NUCELOTIDE ARCHIVE
S. pneumoniae
n = 47,529
FIGURE 1
SEROTYPE 11A
n = 96
WGS
Penicillin Resistant Serotype 11A
Illumina Libraries. Miseq 2x150 bp
1.DOWNLOAD
getSeqENA
github.com/B-UMMI/getSeqENA
2.SEROTYPING
seroBA
github.com/sanger-pathogens/seroba
3.MLST
MLST
github.com/tseemann/mlst
n = 21
France n = 17
Italy n = 3
Portugal n = 4
Spain n = 16
ENA GENOMES
Serotype 11A/9V
ST: 156, SLV, DLV
N = 238
TABLE S2
1.MOLECULAR ANTIBIOTIC RESISTANCE MECHANISM
1.GENE MUTATIONS
Geneious R9
pbp1a, pbp2x, pbp2b, parC, parE, gyrA, folA and folP
from R6 genome (NC_003098)
2.ACQUIRED RESISTANCE
Abricate
CARD and ResFinder
https://github.com/tseemann/abricate
FIGURE S3
TABLE S3
STUDY GENOMES
Penicillin Resistant
Serotype 11A
N = 61
TABLE S1
FIGURE S2
1.ASSEMBLY
INNUca
github.com/B-UMMI/INNUca
2.ANNOTATION
PROKKA
github.com/tseemann/prokka
ALL GENOMES
ENA genomes
Study genomes
N = 299
2.PHYLOGENETIC ANALYSIS
1.CORE-GENOME SNPs TREE
Parsnp from Harvest suite
Reference strain: ERR2681167
PMID 25410596
2.VISUALIZATION
Microreact
PMID 28348833
FIGURE 2A
3.CAPSULAR OPERON ANALYSIS
REGION 1 – 3 ANALYSIS
Geneious R9
Region 1 (pbp2x to dexB)
Region 2 (capsular locus)
Region 3 (aliA to pbp1a)
REFERENCE GENOMES IDENTITY
BLASTn
blast.ncbi.nlm.nih.gov/Blast.cgi
FIGURE 2B
TABLE S4
SELECTED GENOMES
n = 120
LINEAGE 1 ST156
n = 14
9V n = 4
11A n = 10
LINEAGE 2 ST166
n = 38
9V n = 2
11A n = 36
LINEAGE 3 ST838/6521
n = 68
9V n = 13
11A n = 55
4.IDENTIFICATION OF RECOMBINATION REGIONS
1.CORE-SNP ALIGNMENT BY READS MAPPING
Snippy
github.com/tseemann/snippy
1.CONCATENTATION OF THE DRAFT GENOME FROM A RANDOM STRAIN
Mauve
Strain ERR2303060 draft genome mapped against
NZ_LS483448 (Closed 9V-ST156 genome)
2.RECOMBINANT REGIONS DETECTION BY DENSISTY BASE SUBSTITUTION
Gubbins
github.com/sanger-pathogens/gubbins
2.MAXIMUM-LIKELIHOOD PHYLOGENETIC TREE
RaxML
Reference strain: Concatenated ERR2303060
PMID 31070718
3.VISUALIZATION
Phandango
PMID
FIGURE 3
4.ACQUIRED FRAGMENT ANALYS
Geneious R9
Region A-D
Region 1-7
5.REFERENCE GENOMES IDENTITY
BLASTn
blast.ncbi.nlm.nih.gov/Blast.cgi
TABLE S5
5.ACCESSORY AND CORE GENOME
1.GENE PRESENCE/ABSENCE
Roary
Region 1 (pbp2x to dexB)
Region 2 (capsular locus)
Region 3 (aliA to pbp1a)
github.com/sanger-pathogens/Roary
2.GENE PRESENCE/ABSENCE ASSOCIATION BY ST
Scoary
github.com/AdmiralenOla/Scoary

Supplementary Figure S2. Geographical distribution of strains subjected to whole genome sequencing. The map shows the region where strains were isolated. A total of 61 strains from 25 regions in 4 countries were included in this study.

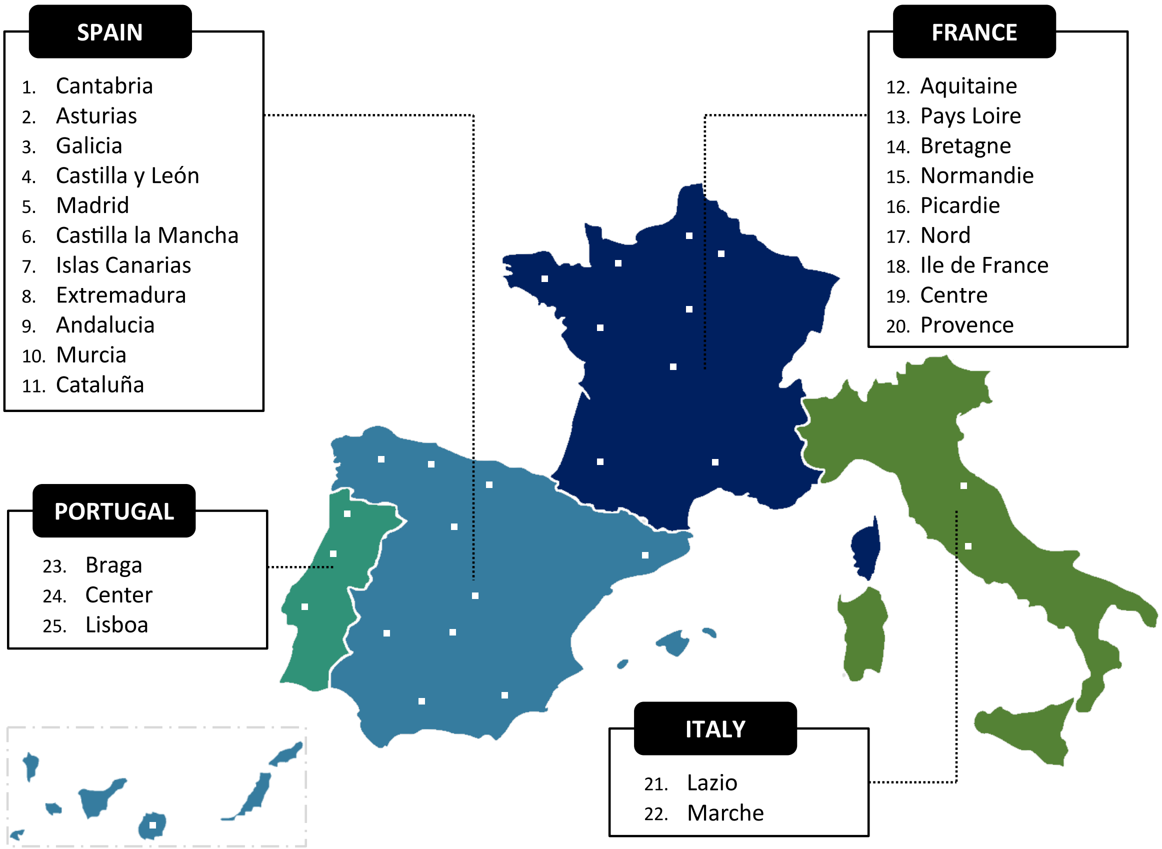

Supplementary Figure S3. Antibiotic and molecular resistance mechanism. Strains are grouped by MIC (mg/L) for penicillin, amoxicillin, cefotaxime, erythromycin, tetracycline, and co-trimoxazole; amino acid changes in the proteins implied in β-lactams resistance (Pbp2X, Pbp1A, Pbp2B); the presence of acquired resistance genes and amino acid changes in the DHFR and DHPS proteins. A bar (-) in DHFR protein represents no amino acid changes compared with R6 strains. PEN: Penicillin; AMOX: Amoxicillin; CTX: Cefotaxime; ERY: Erythromycin; TET: Tetracycline; SXT: Co-trimoxazole.

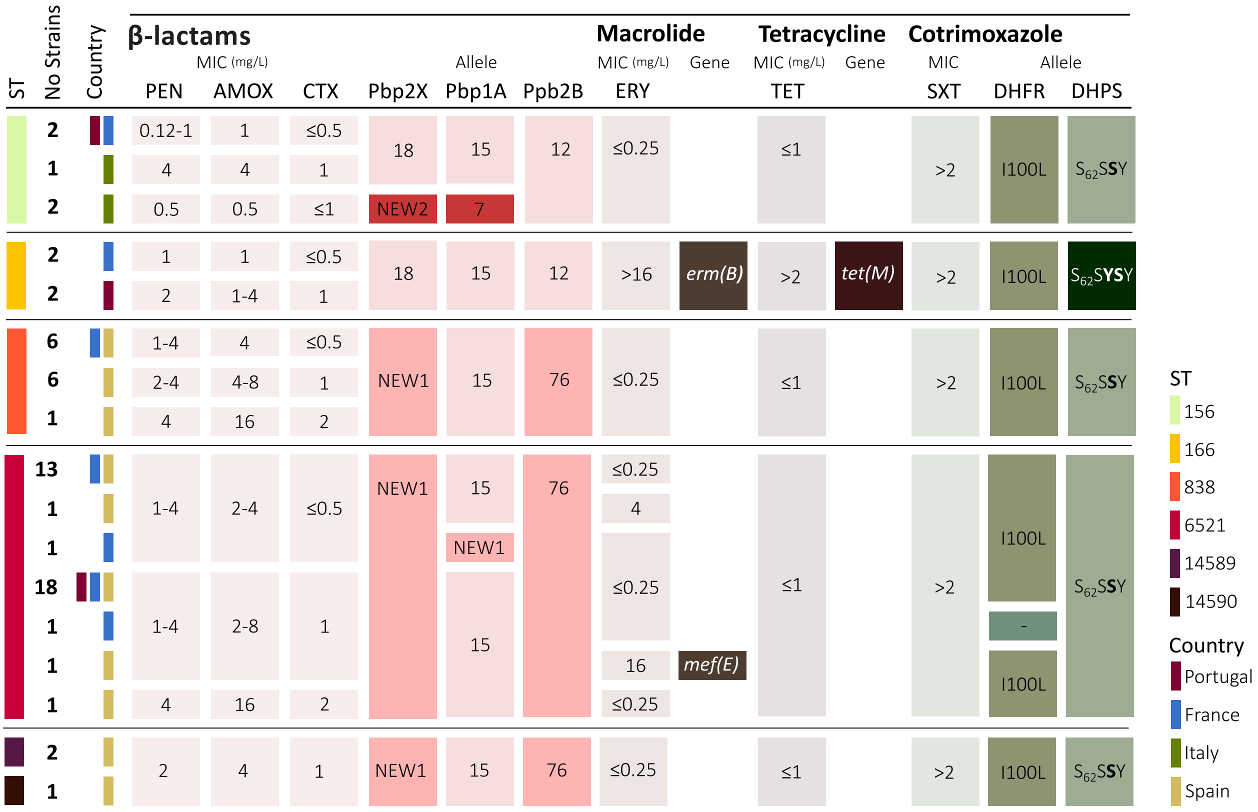

Supplementary Table S1. Demographic, antibiotic MIC and WGS-derivate metadata.

(a) Underlined and boldface ID indicate previously published strains
(b) SP: Spain, IT; Italy, FR; France, PT; Portugal.
(c) Capsular structures are the ones described in Figure 3

Supplementary Table S2. European Nucleotide Archive available genomes. *S. pneumoniae* genomes downloaded on 10-29-2018 belonging to serotype 9V or 11A with ST156, ST166, ST838 or ST6521.

Supplementary Table S3. Protein Alleles. The table summarizes the Pbp2X, Pbp1A, Pbp2B, DHFR and DHPS protein sequences of each strain compared to the R6 reference strain. Polymorphic sites associated with β-lactam resistance are highlighted with blue.

Supplementary Table S4. Amino acid sequence variation in the capsular operon. Amino acid changes relative to the capsular operon of strain 980/60-9V for serotype 9V strains and relative to the capsular operon of strain 1813/39-11A for serotype 11A strains.

Supplementary Table S5. Detailed description of the ORFs present in the recombination regions (core-genome and capsular locus). For each recombinational region in Figure 3 the putative genes are described. For each ORF, the locus tag of the genome-like recipient as well the putative protein function is specified. An approximate coordinates of the close genome 9V-ST156 (4041STDY6836167) are indicated as well the recombinational fragment size. Capsular locus genes are marked in grey, genes where the recombination could have occurred are coloured in light-blue. Those sequences which presented higher identity with the 4041STDY6836167 genome inside the recombinational regions are marked in yellow. Finally, regions inside recombinant fragment which are not related with the genome donor or genome recipient are marked in orange.
